# Contextuality Analysis of Impossible Figures

**DOI:** 10.3390/e22090981

**Published:** 2020-09-03

**Authors:** Víctor H. Cervantes, Ehtibar N. Dzhafarov

**Affiliations:** 1 Department of Psychology, University of Illinois at Urbana-Champaign, Champaign, IL 61820, USA; victorhc@illinois.edu; 2 Department of Psychological Sciences, Purdue University, West Lafayette, IN 47907, USA

**Keywords:** contextuality, deterministic systems, epistemic probabilities, impossible figures, measures of contextuality

## Abstract

This paper has two purposes. One is to demonstrate contextuality analysis of systems of epistemic random variables. The other is to evaluate the performance of a new, hierarchical version of the measure of (non)contextuality introduced in earlier publications. As objects of analysis we use impossible figures of the kind created by the Penroses and Escher. We make no assumptions as to how an impossible figure is perceived, taking it instead as a fixed physical object allowing one of several deterministic descriptions. Systems of epistemic random variables are obtained by probabilistically mixing these deterministic systems. This probabilistic mixture reflects our uncertainty or lack of knowledge rather than random variability in the frequentist sense.

## 1. Introduction

Our main purpose is to illustrate the use of epistemic random variables using objects that are naturally described in a deterministic way, but not uniquely. That is, these objects are described by one of several deterministic systems. The first applications of contextuality analysis to such systems is presented in Reference [1], using various deterministic representations of the Liar’s paradox. In the present paper the objects of the analysis are the so-called impossible figures: the Penrose triangle and several similar figures, as well as the Ascending and Descending staircase lithograph by M. C. Escher. The triangle and the staircase figures were famously discussed by Penrose and Penrose [2]. In Appendix B to this paper we report several measures of contextuality computed for our systems of epistemic random variables, but in the main text we focus on one measure only, introduced here for the first time, a hierarchical version of the (non)contextuality measure ${\mathrm{CNT}}_{2}$-${\mathrm{NCNT}}_{2}$ described in References [3,4].

The Contextuality-by-Default (CbD) theory [1,3,4,5] has been developed to apply to abstract systems of random variables, irrespective of one’s interpretation of probabilistic notions involved. However, most of its applications have dealt with empirical data and random variables understood in the frequentist sense. In this paper we use the term *epistemic random variable* to denote a variable for which the probabilities with which it falls in different sets of possible values reflect our uncertainty or lack of knowledge rather than random variability in the frequentist sense.

A system R of random variables is a set of double-indexed random variables $R_{q}^{c}$, where q∈Q denotes their *content*, that can be defined as a question to which the random variable responds, and c∈C is their *context*, encompassing the conditions under which it is recorded. A system can be presented as

(1) $$R=\{R_{q}^{c}:c\in C,q\in Q,q≺c\},$$

where q≺c indicates that content *q* is responded to in context *c*. The variables of the subset

(2) $$R^{c}=\left\{R_{q}^{c}:q\in Q,q≺c\right\}$$

are *jointly distributed*, whereas any two random variables in the subset

(3) $$R_{q}=\left\{R_{q}^{c}:c\in C,q≺c\right\}$$

are *stochastically unrelated.* (More generally, any two $R_{q}^{c},R_{q^{\prime }}^{c^{\prime }}\in R$ with $c\neq c^{\prime }$ are stochastically unrelated. That is, they do not possess a joint distribution.) The set $R^{c}$ is called the *bunch* corresponding to context *c* and the set $R_{q}$ is referred to as the *connection* for content *q*.

We will limit our discussion and applications to finite systems of binary random variables, with $n=\left|Q\right|$ and $m=\left|C\right|$. Without loss of generality we may assume all variables $R_{q}^{c}$ take values 0/1. These systems can be represented by three vectors. The first one is

(4) $$\begin{array}{ccc} l & = & {\left(1,p_{q}^{c}:q\in Q,c\in C,q≺c\right)}^{⊺}, \end{array}$$

the vector of the low-level marginals, $p_{q}^{c}=\langle R_{q}^{c}\rangle =\mathrm{Pr}\left(R_{q}^{c}=1\right)$, with 1 formally equal to 〈·〉. The second vector is

(5) $$\begin{array}{ccc} c & = & (\min\left(p_{q}^{c_{1}},p_{q}^{c_{2}}\right):q\in Q,c_{1},c_{2}\in C,q≺c_{1},c_{2},c_{1}\neq c_{2}), \end{array}$$

the vector of maximal probabilities with which two random variables in a connection could both equal 1 (if they possessed a joint distribution). It is uniquely determined by vector l. The third vector is

(6) $$b={\left(b_{2},\ldots ,b_{r}\right)}^{⊺},$$

where

(7) $$b_{s}=\left(p_{q_{1},\ldots ,q_{s}}^{c}:q_{1},\ldots ,q_{s}\in Q,c\in C,q_{1},\ldots ,q_{s}≺c,q_{1},\ldots ,q_{s}\;\mathrm{are}\;\mathrm{distinct}\right).$$

Here, s=2,…,r, with $p_{q_{1},\ldots ,q_{s}}^{c}=\langle R_{q_{1}}^{c}\cdots R_{q_{s}}^{c}\rangle =\mathrm{Pr}\left(R_{q_{1}}^{c}=1,\ldots ,R_{q_{s}}^{c}=1\right)$, and 2≤r≤n is the largest number of distinct *q*’s within a bunch. (We could allow r=1, in which case b is empty and the system is trivially noncontextual.) Thus, b is a minimal set of probabilities that completely describes the joint distributions of the bunches in the system (for a given vector l). The coordinates of a specific system of random variables are then given by

(8) $$p^{*}={\left(l^{*},c^{*},b^{*}\right)}^{⊺}.$$

This is the *reduced vectorial representation* of the system, as discussed in Reference [3]. The system is noncontextual if and only if there is a vector h≥0 (component-wise) such that

(9) $$\mathrm{Mh}=p^{*}.$$

Here, the elements of h are probabilities assigned to all possible combinations of values (1’s and 0’s) assigned to all random variables $R_{q}^{c}$ in the system, and M is an incidence (Boolean) matrix [3,5]: in the row of M corresponding to a given element of $p^{*}$, say, $\mathrm{Pr}(R_{q_{1}}^{c}=\ldots =R_{q_{s}}^{c}=1)$, we put 1 in the columns corresponding to the elements of h in which $R_{q_{1}}^{c},\ldots ,R_{q_{s}}^{c}$ are assigned the value 1; other columns in this row are filled with 0.

Denoting the rows of M that correspond to $l^{*},c^{*},b^{*}$ by, respectively, $M_{l},M_{c},M_{b}$, we can rewrite (9) in extenso:

(10) $${\left(M_{l},M_{c},M_{b}\right)}^{⊺}h={\left(l^{*},c^{*},b^{*}\right)}^{⊺}.$$

Reference [3] also introduces a measure of contextuality, ${\mathrm{CNT}}_{2}$, with its natural extension into a measure of noncontextuality, ${\mathrm{NCNT}}_{2}$. In Reference [4], the behavior of both measures was characterized for a special class of systems, known as cyclic. These measures can be computed with the aid of linear programming. To compute ${\mathrm{CNT}}_{2}$, one solves the following task

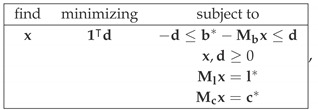



And, for any solution $x^{*}$, one finds ${\mathrm{CNT}}_{2}={∥b^{*}-M_{b}x^{*}∥}_{1}$ ($L_{1}$-norm). By enumerating the elements of $b^{*}$ as 1,…,K, the ${\mathrm{NCNT}}_{2}$ measure of noncontextuality is computed as

(11) $${\mathrm{NCNT}}_{2}=\min_{i=1,\ldots ,K}\left\{\min\left(d_{i}^{*-},d_{i}^{*+}\right)\right\},$$

where $d_{i}^{*-},d_{i}^{*+}$ are solutions of the following linear programming tasks (denoting by $e_{i}$ the unit vector with unity in its *i*-th element)

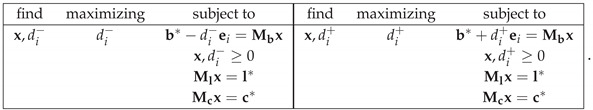



Clearly, for a contextual system ${\mathrm{CNT}}_{2}>0$ and ${\mathrm{NCNT}}_{2}=0$, whereas for a noncontextual one, ${\mathrm{CNT}}_{2}=0$ and ${\mathrm{NCNT}}_{2}\geq 0$.

## 2. Hierarchically Measuring Contextuality

The development of this measure has greatly benefited from discussions with and critical analysis by Janne V. Kujala (see Acknowledgements).

For a given vector $l^{*}$ (hence also the vector $c^{*}$), we call the convex polytope

(12) $$K=\{b|\exists h\geq 0:{\left(M_{l},M_{c},M_{b}\right)}^{⊺}h={\left(l^{*},c^{*},b\right)}^{⊺}\}$$

the *noncontextuality* polytope. A system represented by the point $p^{*}={\left(l^{*},c^{*},b^{*}\right)}^{⊺}$ is noncontextual if and only if $b^{*}\in K$. The measures ${\mathrm{CNT}}_{2}$ and ${\mathrm{NCNT}}_{2}$ are the $L_{1}$-distance from $b^{*}$ to the surface of K when the system is, respectively, contextual or noncontextual.

In References [4,6] it is noted that

(13) $$\max\left(0,p_{q_{1}}^{c}+p_{q_{2}}^{c}-1\right)\leq p_{q_{1},q_{2}}^{c}\leq \min\left(p_{q_{1}}^{c},p_{q_{2}}^{c}\right).$$

This observation is easily generalized as

(14) $$\max\left(0,\frac{p_{q_{1},q_{2}}^{c}+p_{q_{1},q_{3}}^{c}+p_{q_{2},q_{3}}^{c}-1}{2}\right)\leq p_{q_{1},q_{2},q_{3}}^{c}\leq \min\left(p_{q_{1},q_{2}}^{c},p_{q_{1},q_{3}}^{c},p_{q_{2},q_{3}}^{c}\right),$$

(15) $$\begin{array}{c} \max\left(0,\frac{p_{q_{1},q_{2},q_{3}}^{c}+p_{q_{1},q_{2},q_{4}}^{c}+p_{q_{1},q_{3},q_{4}}^{c}+p_{q_{2},q_{3},q_{4}}^{c}-1}{3}\right)\leq p_{q_{1},q_{2},q_{3},q_{4}}^{c}\\ \leq \min(p_{q_{1},q_{2},q_{3}}^{c},p_{q_{1},q_{2},q_{4}}^{c},p_{q_{1},q_{3},q_{4}}^{c},p_{q_{2},q_{3},q_{4}}^{c}), \end{array}$$

and in complete generality,

(16) $$\begin{array}{c} \max\left(0,\frac{1}{s-1}\left(\sum_{k=1}^{s}p_{\left\{q_{1},\ldots ,q_{s}\right\}\backslash \left\{q_{k}\right\}}^{c}-1\right)\right)\leq p_{q_{1},\ldots ,q_{s}}^{c}\\ \leq \min(p_{\left\{q_{1},\ldots ,q_{s}\right\}\backslash \left\{q_{k}\right\}}^{c}:k=1,\ldots ,s), \end{array}$$

for s=2,…,r. That is, the elements of $b^{*}$ are hierarchically bounded, with $l^{*}$ providing the bounds for $b_{2}^{*}$ and $b_{s-1}^{*}$ determining the bounds for $b_{s}^{*}$ if 2<s≤r.

These hierarchical restrictions suggest a hierarchical way of approaching the measurement of the (non)contextuality of a system R represented by $p^{*}$. Consider the systems of equations

(17) $$\begin{array}{ccc} {\left(M_{l},M_{c},M_{2}\right)}^{⊺}h_{2} & = & {\left(l^{*},c^{*},b_{2}^{*}\right)}^{⊺}\\ & \vdots \\ {\left(M_{l},M_{c},M_{2},\ldots ,M_{s}\right)}^{⊺}h_{s} & = & {\left(l^{*},c^{*},b_{2}^{*},\ldots ,b_{s}^{*}\right)}^{⊺}\\ & \vdots \\ {\left(M_{l},M_{c},M_{2},\ldots ,M_{r}\right)}^{⊺}h_{r} & = & {\left(l^{*},c^{*},b_{2}^{*},\ldots ,b_{r}^{*}\right)}^{⊺}={\left(l^{*},c^{*},b^{*}\right)}^{⊺}=Mh, \end{array}$$

where $M_{s}$ is the submatrix formed by the rows of M corresponding to the elements of $b_{s}^{*}$. Clearly, for a contextual system there is a value $2\leq s^{*}\leq r$ such that there is no solution $h_{s}\geq 0$ for any $s\geq s^{*}$ while there is a solution for each $s<s^{*}$. For a noncontextual system, the solution to the system (9) implies the solution to all systems (17). Therefore, if R is contextual, we can further qualify its contextuality and say that it is contextual at level $s^{*}$. Its degree of contextuality at level $s^{*}$ can be computed by solving the linear programming task

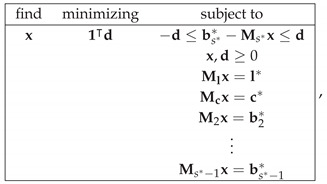



And computing, for any solution $x^{*}$,

(18) $${\mathrm{CNT}}_{2}^{s^{*}}={∥b_{s^{*}}^{*}-M_{s^{*}}x^{*}∥}_{1}.$$

Moreover, for each *s* for which ${\left(M_{l},M_{c},M_{2},\ldots ,M_{s}\right)}^{⊺}h_{s}={\left(l^{*},c^{*},b_{2}^{*},\ldots ,b_{s}^{*}\right)}^{⊺}$ has a solution, we can compute the noncontextuality of R at level *s* as

(19) $${\mathrm{NCNT}}_{2}^{s}=\min_{i=1,\ldots ,K_{s}}\left\{\min\left(d_{i}^{*-},d_{i}^{*+}\right)\right\},$$

where $K_{s}$ is the number of elements of $b_{s}^{*}$, and $d_{i}^{*-},d_{i}^{*+}$ are solutions to the linear programming tasks

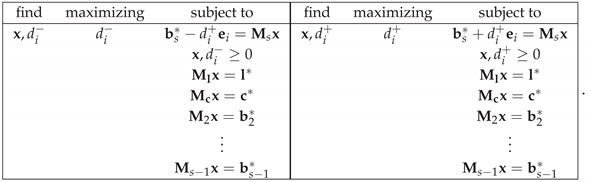



In this way, we construct the hierarchical measure of (non)contextuality which characterizes the degree of (non)contextuality of a system R by a vector of size $s^{*}-1$ if the system is contextual, or of size r−1 if the system is noncontextual. For a contextual system, ${\mathrm{CNT}}_{2}^{s^{*}}$ gives the $L_{1}$-distance from $b_{s^{*}}^{*}$ to the surface of the polytope

(20) $$K_{s^{*}}=\left\{\begin{array}{cc} \{b_{2}|\exists h\geq 0:{\left(M_{l},M_{c},M_{2}\right)}^{⊺}h_{2}=\\ \;\;\;{\left(l^{*},c^{*},b_{2}\right)}^{⊺}\} & \mathrm{if}\;s^{*}=2\\ \{b_{s^{*}}|\exists h\geq 0:{\left(M_{l},M_{c},M_{2},\ldots ,M_{s^{*}}\right)}^{⊺}h_{s^{*}}=\\ \;\;\;{\left(l^{*},c^{*},b_{2}^{*},\ldots ,b_{s^{*}-1}^{*},b_{s^{*}}\right)}^{⊺}\} & \mathrm{if}\;2<s^{*}\leq r \end{array}\right..$$

And, at each level $2\leq s<s^{*}$ (2≤s≤r for a noncontextual system), ${\mathrm{NCNT}}_{2}^{s}$ is the $L_{1}$-distance from $b_{s}^{*}$ to the surface of the polytope

(21) $$K_{s}=\left\{b_{s}|\exists h\geq 0:{\left(M_{l},M_{c},M_{2},\ldots ,M_{s}\right)}^{⊺}h_{s}={\left(l^{*},c^{*},b_{2}^{*},\ldots ,b_{s-1}^{*},b_{s}\right)}^{⊺}\right\}.$$

Analogously to ${\mathrm{CNT}}_{2}$ and ${\mathrm{NCNT}}_{2}$, we have that for a system contextual at level $s^{*}$, ${\mathrm{CNT}}_{2}^{s^{*}}>0$ and ${\mathrm{NCNT}}_{2}^{s^{*}}=0$, and in addition for $s<s^{*}$, ${\mathrm{CNT}}_{2}^{s}=0$.

## 3. The Penrose Triangle

We will now apply the measures just constructed to some drawings known as impossible figures. The general idea underlying their contextuality analysis is that the epistemic random variables representing an impossible figure should always be compared to those representing a realizable (i.e., “normal”) figure, and the degree of contextuality be derived from the difference between the two.

**Figure 1:**
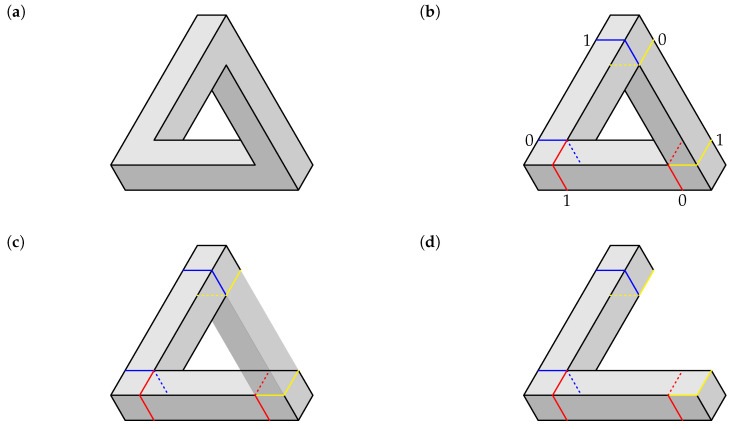
(**a**) The Penrose triangle. (**b**) The Penrose triangle with superimposed cuts, shown with the corresponding labels (0 or 1). (**c**) Look through the right bar of the triangle. (**d**) Right bar removed by its cuts.

**Figure 2:**
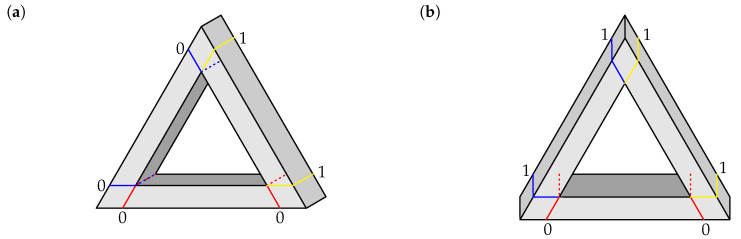
Realizable triangles with superimposed cuts, shown with the corresponding labels (0 or 1). (**a**) Triangle at 30 deg oblique projection. (**b**) Triangle at 90 deg oblique projection.

We begin with the well-known Penrose triangle, depicted in Figure 1a. Observe that one can see precisely two faces of each of the three bars forming the figure. So each corner of the triangle is formed by four of these faces, one of which ends in the inner fold of the corner (invisible in Figure 1a; in other figures, for example, in Figure 2a, if the inner fold is visible, then two faces, of two different bars, end in it). Looking at the two faces of a given bar near one of the corners, either one of the faces ends in the inner fold while another does not (we encode this case by 0), or both do not (encoded as 1). In Figure 1b this is shown by interrupted and solid lines of the cuts made in each bar at the two corners it connects. The endpoint labels 1 and 0 correspond to, respectively, the case when both cut lines are solid, and the case when one of them is interrupted.

We do not claim that this encoding describes how the figure or its elements are perceived. The latter is a question for an empirical investigation, such as the one presented in Reference [7], where contextuality analysis was applied to perception of an ambiguous figure (Schröder’s stair). We are merely selecting a possible description of the figure as a fixed physical object. Other mathematical descriptions of the Penrose triangle and other impossible figures (some of them very different from those considered in this paper) can be found in References [8,9,10].

Perception may make use of such descriptions, but it is most likely a complex process with descriptions changing in time.

We can construct a system representation of the Penrose triangle in two ways. The first one consists in looking at each of the three bars separately, and arbitrarily picking one of its two ends as the first one in an ordered pair. We see in Figure 1 that whenever the end we pick is coded 0, the other end of the bar is coded 1, and vice-versa. Thus, we get two deterministic descriptions,
(22)
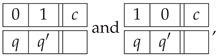

where *c* designates a specific bar (here, left, right, or bottom one), and $\left(q,q^{\prime }\right)$ is an ordered pair of its endpoints. Since these deterministic descriptions are equally plausible (i.e., there is no preferred ordering of the endpoints of an isolated bar), we can assign the epistemic probability 1/2 to each of them, obtaining thereby two perfectly anticorrelated uniformly distributed epistemic random variables,

(23) $$\begin{array}{cccc} \; & R_{q^{\prime }}^{c}=0 & R_{q^{\prime }}^{c}=1\\ R_{q}^{c}=0 & 0 & 1\;/\;2 & 1\;/\;2\\ R_{q}^{c}=1 & 1\;/\;2 & 0 & 1\;/\;2\\ & 1\;/\;2 & 1\;/\;2 \end{array}\;.$$

The stand-alone numbers in the table are joint and marginal probabilities.

We now have to add for comparison epistemic random variables describing a bar of a realizable (i.e., “normal”) triangle, as the ones shown in Figure 2. We define a realizable figure as one that can be viewed as an oblique projection of a physical object of relatively small thickness (so that perspective can be ignored). Each of the contexts representing a realizable bar has two identically labelled ends, and depending on the oblique projection angle, each bar (left, right, or bottom) can be labeled $\left(1,1\right)$ or $\left(0,0\right)$. Using the same uniform epistemic mixing as before, we obtain two perfectly correlated uniformly distributed epistemic random variables,

(24) $$\begin{array}{cccc} \; & R_{q^{\prime }}^{c}=0 & R_{q^{\prime }}^{c}=1\\ R_{q}^{c}=0 & 1\;/\;2 & 0 & 1\;/\;2\\ R_{q}^{c}=1 & 0 & 1\;/\;2 & 1\;/\;2\\ & 1\;/\;2 & 1\;/\;2 \end{array}\;.$$

Repeating this reasoning for each of the three bars, we get the context-content matrix.

*Option 1*

(25)
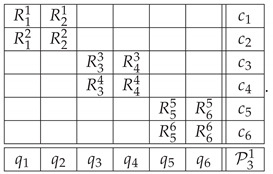

The contexts and contents in $P_{3}^{1}$ have been numbered so that the odd contexts represent the bars of the Penrose triangle (with the perfectly anticorrelated variables), and the even ones represent the corresponding realizable bars (with perfectly correlated variables). This ordering highlights the fact that the system is composed of three disjoint 2×2 subsystems (formally, cyclic systems of rank 2). We call this way of representing the impossible figure *Option 1*.

Another way (*Option 2*) to approach the construction of a system of epistemic random variables describing the Penrose triangle is to look at the sequence of the labels for the cuts in the entire triangle. We arbitrarily choose one of the six cuts in the figure as a starting point, and then proceed to the second cut in the same bar, then to the nearest cut in the adjacent bar, and so forth. This produces one of the two patterns shown below, representing three starting points each:(26)
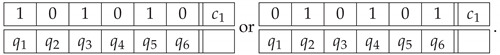

By uniformly mixing these patterns, we obtain a vector of six jointly distributed epistemic random variables that represent the Penrose triangle in a single context of random variables:(27)
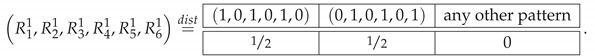

The numbers in the second row are probabilities of the values in the first row, $\overset{dist}{=}$ stands for “distributed as.”

To complete Option 2, we have to add for comparison the epistemic random variables describing a realizable triangle in the same fashion. We select the triangle depictions that may be obtained by oblique projection at arbitrary angles excluding multiples of 60 deg. (We exclude the multiples of 60 deg because at these angles one of the bars is drawn with a single visible side instead of two.) Except for rotations, this process produces two distinct figures with respect to the patterns of 0 and 1 that describe them in a similar way to the Penrose triangle above. The three left patterns in (28) below are the possible patterns that describe the triangle in Figure 2a, and the three patterns on the right describe the one in Figure 2b.
(28)
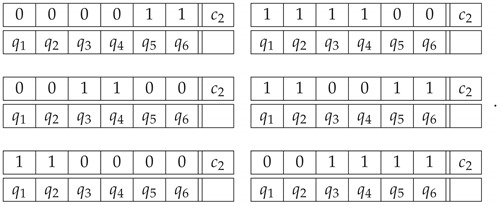

By taking the uniform mixture of these deterministic patterns, we produce a joint distribution that represents a realizable triangle as the second context in the context-content matrix $P_{3}^{2}$ below

*Option 2*

(29)
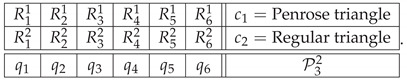



Whichever of the two options we choose, the pairs of random variables that represent two cuts on the same bar are perfectly negatively correlated in the case of the Penrose triangle, and are perfectly positively correlated in a realizable triangle. Because of this the systems in both Options 1 and 2 are contextual. In both cases the contextuality is achieved at level 2. Therefore their hierarchical representation is a one-component vector. The values are ${\mathrm{CNT}}_{2}^{2}=1.5$ in Option 1 and ${\mathrm{CNT}}_{2}^{2}=4.5$ in Option 2. (At this introductory stage we have not discussed the issue of normalization of the (non)contextuality values, because of which we should avoid comparing the values computed for systems of different format, such as our Option 1 and Option 2 systems.)

The value of ${\mathrm{CNT}}_{2}^{2}$ for Option 1 is clearly the sum of the contextuality values of the three disjoint subsystems. Generally, for a system R which is composed of *N* disjoint systems $R_{i}$, the noncontextuality polytope

(30) $$K=\prod_{i=1}^{N}K_{i},$$

the Cartesian product of the corresponding noncontextuality polytopes $K_{i}$ of each system. Consequently,

(31) $${\mathrm{CNT}}_{2}^{s^{*}}\left(R\right)=\sum_{i=1}^{N}{\mathrm{CNT}}_{2}^{s^{*}}\left(R_{i}\right),$$

and

(32) $${\mathrm{NCNT}}_{2}^{s}\left(R\right)=\min_{i=1}^{N}\left\{{\mathrm{NCNT}}_{2}^{s}\left(R_{i}\right)\right\},$$

for $2\leq s\leq s^{*}$.

## 4. Other Impossible Figures

**Figure 3:**
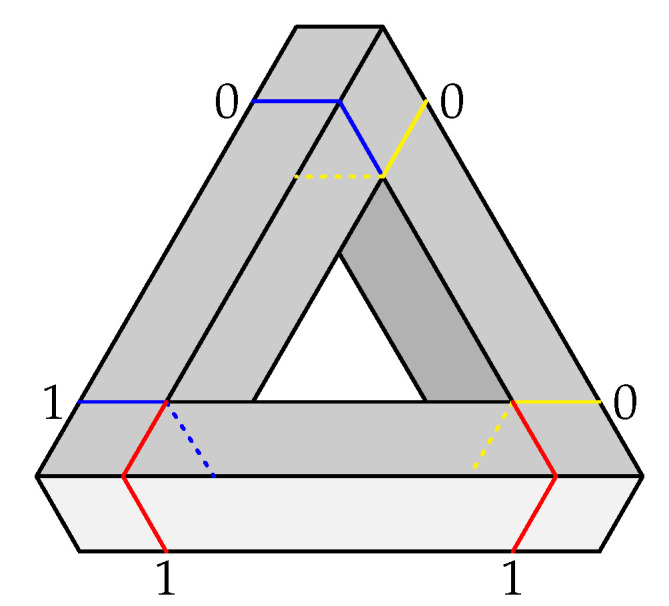
Alternative impossible triangle with cross-sections and corresponding labels (0 or 1).

To further explore how our Option 1 and Option 2 representations capture the impossibility of a figure by the degree of contextuality of the resulting systems, let us consider an alternative impossible triangle, depicted in Figure 3. Unlike the Penrose triangle, this one has only one bar (the left one) with different labels at its ends. For the bottom bar, both ends are coded as 1, and for the right bar the two ends are coded as 0. Intuitively, this triangle seems “less impossible” than the Penrose one. Following the same Option 1 procedure as before, with the same variables describing a realizable triangle, we obtain the system of the same format as system $P_{3}^{1}$; however, in two of the disjoint 2×2 subsystems, in the odd-numbered contexts representing the bottom and right bars, the two random variables become deterministic, making thereby these subsystems noncontextual. (In CbD, any deterministic variable in a system can be deleted from the system without affecting its (non)contextuality, and the same is true for any variables that is alone in its connection.) Therefore, two of the subsystems, corresponding to the lower and the right bars, can be removed without affecting the analysis). For Option 2, we obtain a system of the same format as $P_{3}^{2}$, but the distribution of the bunch corresponding to $c_{1}$ (impossible triangle) is given by the uniform mixture of the patterns below:(33)
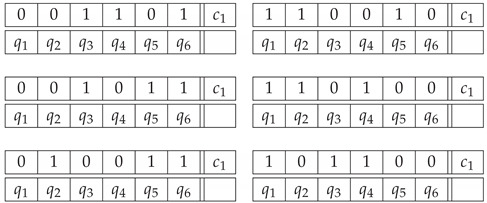

The deterministic patterns whose mixture produces random variables variables in context $c_{2}$ are the same as in (28). The alternative impossible triangle is contextual under both representations, with ${\mathrm{CNT}}_{2}^{2}=0.5$ under Option 1, and ${\mathrm{CNT}}_{2}^{2}=1.5$ under Option 2, both values being lower than the corresponding ones for the Penrose triangle.

Finally, we need to check that the procedures above yield no contextuality if an impossible figure is replaced with a realizable one. We use the realizable triangle in Figure 2a. Option 1 produces three deterministic pairs of random variables for all three odd-numbered contexts corresponding to the pictured triangle. For Option 2, the realizable triangle is represented by a context with the joint distribution given by uniform mixture of the following patterns: (34)
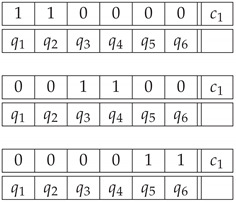

The realizable triangle is noncontextual under both options, and all its hierarchical ${\mathrm{NCNT}}_{2}^{s}$ measures are zero (indicating that the systems for the realizable triangle lie on the surface of the corresponding noncontextuality polytopes).

**Figure 4:**
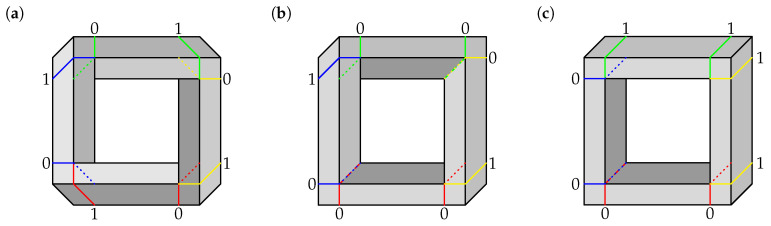
Square figures, shown with cuts and corresponding labels (0 or 1). (**a**) Impossible square. (**b**) Alternative impossible square. (**c**) Realizable “normal” square.

Contextuality analysis similar to the reported above for the impossible triangles can be extended to other impossible figures. We have conducted this analysis for the impossible square (or rectangle) and the impossible circle (also known as impossible loop). For the impossible square, in addition to the figure constructed with the same type of corners as in the Penrose triangle (Figure 4a), we have considered an alternative impossible square (Figure 4b). Figure 4c shows a realizable (“normal”) square. Since the procedures and reasoning here are in all essential details the same as for the impossible triangles, we have relegated the details to Appendix A.

**Figure 5:**
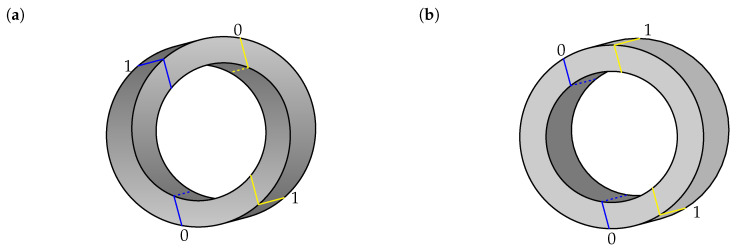
Circular figures, shown with cuts and corresponding labels (0 or 1). (**a**) Impossible circle. (**b**) Realizable circle.

Figure 5 depicts an impossible circle (Figure 5a) and a realizable one (Figure 5b). To characterize the circles, we may look at them as being composed of two handles joined by curved bars, with the cut lines drawn between the handles and the joining bars. In this way, we obtain systems analogous to those we found for the two ways we are using to represent the figures. Again, we relegate the details of the analysis to Appendix A.

We summarize the results of our analysis in Table 1. As we see, all the impossible figures we explored, under both Option 1 and Option 2, are contextual at level 2.

## 5. Escher’s “Ascending and Descending”

**Figure 6:**
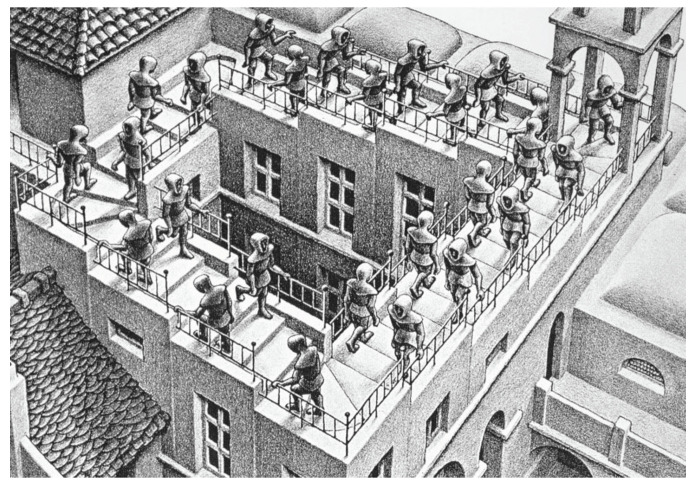
Fragment of M. C. Escher’s lithograph “Ascending and Descending.”

We approach the Ascending and Descending lithograph by M. C. Escher (Figure 6) by considering the four stair flights as four contents, $q_{1},q_{2},q_{3},q_{4}$. The same as for the impossible figures, we have two ways of constructing the system of epistemic random variables. In Option 1, the system is represented by the following context-content matrix:

*Option 1*

(35)
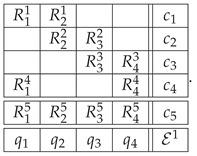



If we view a stair flight as ascending, then $R_{q}^{c}=1$, otherwise $R_{q}^{c}=0$. For every pair of consecutive stair flights in the picture, they are seen as both ascending, or both descending. By uniformly mixing
(36)
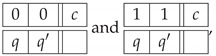

we form the first four contexts in the system. Thus, in contexts $c_{1}$ to $c_{4}$, the distributions are described by

(37) $$\langle R_{k}^{k}\rangle =\langle R_{k⊕1}^{k}\rangle =0.5,\langle R_{k}^{k}R_{k⊕1}^{k}\rangle =1,$$

for k=1,…,4 (⊕ is cyclic addition, with 4⊕1=1). The fifth context in $E^{1}$ includes the possible patterns of ascent and descent for the entire staircase. The strangeness (or impossibility) of the situation in the lithograph is that four stair flights forming a closed loop cannot ascend or descend indefinitely: the number of ascending stair flights should be precisely two to counterbalance the descending ones. In other words, the physically possible values of the bunch $\left(R_{1}^{5},R_{2}^{5},R_{3}^{5},R_{4}^{5}\right)$ are
(38)
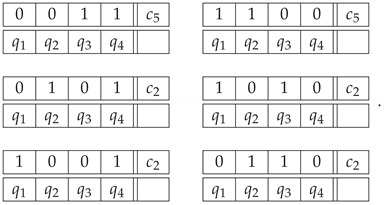

The epistemic distribution of the fifth bunch therefore is given by the uniform mixture of these deterministic patterns.

The second way in which we represent the Ascending-Descending staircase is by looking at the four stair flights together. This forms one (“impossible”) context where either all staircases are described as ascending ($R_{q}^{1}=1,\;\mathrm{for}\;q=1,\ldots ,4$) or all of them are descending ($R_{q}^{1}=0,\;\mathrm{for}\;i=1,\ldots ,4$). A second context, describing the physically realizable patterns is formed in the same way as the fifth context above. The system we obtain in this way is

*Option 2*

(39)
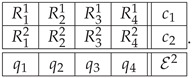

For both these options, the resulting systems are contextual at the level $s^{*}=2$, and the values are ${\mathrm{CNT}}_{2}^{2}=4\;/\;3$ for Option 1 and ${\mathrm{CNT}}_{2}^{2}=2$ for Option 2.

## 6. Conclusions

We have introduced a hierarchical measure of (non)contextuality of systems of random variables. It follows the same logic and is calculated similarly to the measures of contextuality ${\mathrm{CNT}}_{2}$ and noncontextuality ${\mathrm{NCNT}}_{2}$. It is clear that in cyclic systems the hierarchical measure equals ${\mathrm{CNT}}_{2}$ when the system is contextual, and it equals ${\mathrm{NCNT}}_{2}$ when the system is noncontextual. It still remains to investigate whether some of the properties of ${\mathrm{CNT}}_{2}$-${\mathrm{NCNT}}_{2}$ described in Reference [4] for cyclic systems generalize to some classes of noncyclic systems.

The analysis of the impossible figures show that the intuitive degree of the impossibility or strangeness of those figures can be captured through the contextuality of the systems of epistemic random variables chosen to describe them. When the endpoint codes of a bar in a Penrose-like figure are anticorrelated, the adjacent bars appear to twist toward different directions. The more such “twisted” situations we see in a figure, the stranger it looks, and the greater its contextuality (if achieved at the same level).

All contextual systems constructed in this paper happen to be contextual at level 2. This cannot be otherwise for the Option 1 systems which only contain two random variables in each bunch. However, it is an empirical rather than mathematically deducible fact for other systems. More work is needed to find out the scope of the impossible figures whose reasonable descriptions have the same property.

## Figures and Tables

**Table 1:** Measures of contextuality for the impossible figures.

Impossible Figure	$\begin{array}{c} \mathrm{Option}1\\ {\mathrm{CNT}}_{2}^{2} \end{array}$	$\begin{array}{c} \mathrm{Option}2\\ {\mathrm{CNT}}_{2}^{2} \end{array}$
Triangle in Figure 1	1.5	4.5
Triangle in Figure 3	0.5	1.5
Square in Figure 4a	2.0	8.0
Square in Figure 4b	1.0	2.0
Circle in Figure 5a	1.0	2.0

## Appendix A. Impossible Squares and Circles

To create the epistemic random variables depicting realizable (“normal”) square, we proceed analogously to the realizable triangles: we systematically consider all depictions that may be obtained by oblique projection at arbitrary angles excluding the multiples of 90 deg. In this case, ignoring rotations, there is only one pattern of endpoint labels produced by all realizable squares. Varying the starting point, we get the following sequences whose uniform mixture yields the epistemic random variables for realizable squares:

(A1)

All the results for impossible squares are obtained as a straightforward expansion of those for impossible triangles. Following Option 1, we obtain the systems describing the rectangles in Figure 4 in format

*Option 1*

(A2)

The systems for Option 2 have the format

*Option 2*

(A3)

For the first option, just as with the triangles, all random variables representing a realizable bar, are perfectly correlated. The random variables representing bars of the Penrose-like square are negatively correlated, whereas for the alternative impossible square, two of the four odd-numbered bunches become deterministic. For the realizable square in Figure 4c, all four odd-numbered bunches are deterministic, making the system trivially noncontextual.

For Option 2, the patterns that generate the joint distribution for the context describing the Penrose-like impossible square are

(A4)

The patterns that generate the joint distribution for the context describing the alternative impossible square are

(A5)

The measures of contextuality for the impossible square (Figure 4a) are ${\mathrm{CNT}}_{2}^{2}=2$ and ${\mathrm{CNT}}_{2}^{2}=8$, under Options 1 and 2, respectively. The corresponding measures for the alternative impossible square (Figure 4b) are ${\mathrm{CNT}}_{2}^{2}=1$ and ${\mathrm{CNT}}_{2}^{2}=2$. The realizable square (Figure 4c) is noncontextual and all its noncontextuality measures are zero.

For the impossible circle, the system for Option 1 has the format

*Option 1*

(A6)

and the system for the Option 2 has the format

*Option 2*

(A7)

The distributions of the random variables in each context for Option 1 follows the same pattern as the corresponding Option 1 for the triangles and the squares. The two variables in a bunch corresponding to the impossible loop are anticorrelated and the ones corresponding to the realizable one are perfectly correlated. For Option 2,

(A8)

gives the patterns for the impossible circle. The patterns that generate the joint distribution for the bunch representing the realizable circle are

(A9)

The measures of contextuality for the impossible loop are ${\mathrm{CNT}}_{2}^{2}=1$ and ${\mathrm{CNT}}_{2}^{2}=2$, under Options 1 and 2, respectively. The realizable figure is noncontextual and all its noncontextuality measures are zero.

## Appendix B. Measures of Contextuality

For completeness and comparison purposes, we present the values of the contextuality measures ${\mathrm{CNT}}_{1}$, ${\mathrm{CNT}}_{2}$, ${\mathrm{CNT}}_{2}^{2}$, ${\mathrm{CNT}}_{3}$, and contextual fraction (CNTF), for each of the systems used to represent the impossible figures. The measure ${\mathrm{CNT}}_{2}^{2}$ is the hierarchical measure of (non)contextuality described in this paper. The contextual fraction was introduced in Reference [11]. The other measures and the linear programming tasks that may be used to compute them are presented in Reference [3].

Table A1 presents the contextuality measures for the systems representing the triangles. Table A2 presents the contextuality measures for squares figures. Table A3 presents the contextuality measures for the systems representing the two circles. Table A4 presents the contextuality measures of the systems representing Escher’s Ascending and Descending Staircase.

**Table A1:** Contextuality measures of systems representing the alternative impossible triangle and a realizable triangle.

Figure	System	${\mathrm{CNT}}_{1}$	${\mathrm{CNT}}_{2}$	${\mathrm{CNT}}_{2}^{2}$	${\mathrm{CNT}}_{3}$	CNTF
Penrose triangle	Option 1	1.5	1.5	1.5	1	1
	Option 2	1.5	8.0	4.5	1	1
Alt. Imp.	Option 1	0.5	0.5	0.5	1	1
	Option 2	0.5	3.0	1.5	1/3	1
Realizable	Option 1	0	0	0	0	0
	Option 2	0	0	0	0	0

**Table A2:** Contextuality measures of systems representing the square figures.

Figure	System	${\mathrm{CNT}}_{1}$	${\mathrm{CNT}}_{2}$	${\mathrm{CNT}}_{2}^{2}$	${\mathrm{CNT}}_{3}$	CNTF
Penrose like	Option 1	2	2	2	1	1
	Option 2	2	18	8	1	1
Alt. Imp.	Option 1	1	1	1	1	1
	Option 2	1	2	2	1/2	1
Realizable	Option 1	0	0	0	0	0
	Option 2	0	0	0	0	0

**Table A3:** Contextuality measures of systems representing the circular figures.

Figure	System	${\mathrm{CNT}}_{1}$	${\mathrm{CNT}}_{2}$	${\mathrm{CNT}}_{2}^{2}$	${\mathrm{CNT}}_{3}$	CNTF
Impossible	Option 1	1	1	1	1	1
	Option 2	1	2	2	1	1
Realizable	Option 1	0	0	0	0	0
	Option 2	0	0	0	0	0

**Table A4:** Contextuality measures of systems representing the Ascending and Descending staircase.

Representation	${\mathrm{CNT}}_{1}$	${\mathrm{CNT}}_{2}$	${\mathrm{CNT}}_{2}^{2}$	${\mathrm{CNT}}_{3}$	CNTF
Option 1	6/3	4/3	4/3	2/3	1
Option 2	1	9/2	2	1	1
